# Lipopolysaccharide Effects on Neurotransmission: Understanding Implications for Depression

**DOI:** 10.1021/acschemneuro.4c00591

**Published:** 2024-11-27

**Authors:** L. Batey, B. Baumberger, H. Khoshbouei, P. Hashemi

**Affiliations:** ∇ Department of Bioengineering, Imperial College, South Kensington, London, SW7 2AZ, U.K.; ‡ Department of Neuroscience, University of Florida College of Medicine, Gainesville, Florida 32610, United States

**Keywords:** inflammation, microglia, neurotransmission, cytokines, norepinephrine, serotonin

## Abstract

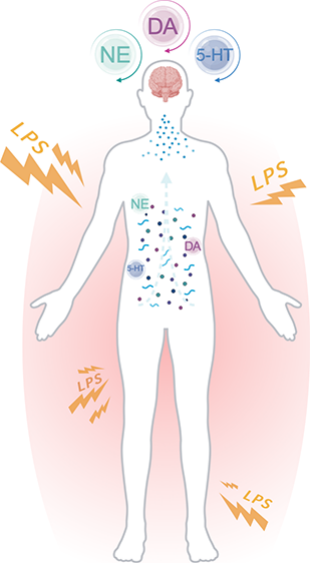

Immune activation in the body is well studied; however, much less is known about how peripheral inflammation changes brain chemistry. Because depression and inflammation are close comorbidities, investigating how inflammation affects the brain’s chemicals will help us to better understand depression. The levels of the monoamines dopamine, serotonin and norepinephrine are thought to be affected by both inflammation and depression. In this Perspective, we review studies that find chemical changes in the brain after administration of the endotoxin LPS, which is a robust method to induce rapid inflammation. From these studies, we interpreted LPS to reduce dopamine and serotonin and increase norepinephrine levels in various regions in the brain. These changes are not a sign of “dysfunction” but serve an important evolutionary purpose that encourages the body to recover from an immune insult by altering mood.

## Introduction

Inflammation is a blanket term used to describe the body’s immune response to an irritant. Immune activation in the periphery serves important physiological purposes (*i.e*., elimination of pathogens/foreign bodies) via biochemical cascades involving several cell types, that are relatively well established.^1^ In recent times, the literature supports a strong connection between body and brain inflammation.^2^ However, the effect of inflammation on brain chemistry is much less understood. What is clear is that inflammation and mental health disorders, particularly depression, exhibit significant comorbidity.

In addition to the comorbidity between depression and inflammation, there is a significant or full overlap between sickness and depressive behaviors.^3,4^ Therefore, researchers are studying the underlying pathology of inflammation to better explain depression.^5^ To this end, observing the detailed effects of inflammation on neurotransmitters, which control mood, would be highly informative.

The neurotransmitters that drive mood likely involve the neuromodulators dopamine (DA), serotonin and norepinephrine (NE).^6−8^ These modulators are thought to be involved because of a long-standing notion arising from the monoamine hypothesis of depression (that the levels of DA, serotonin and NE are lower during depression). While this hypothesis has cycled in and out of popularity for decades, a rich body of literature supports how monoamines drive affective behaviors.^9,10^ Compellingly, there is also extensive literature to support a profound effect of inflammation on these monoamines (reviewed in this perspective).

It is difficult to select an appropriate model to study how inflammation orchestrates monoamine concentrations because immune system activation is complex, heterogeneous, and comorbid with many other brain disorders such as Parkinson’s disease and autism spectrum disorder.^11,12^ A robust and well-studied way to induce rapid, acute inflammation is via introduction of lipopolysaccharide (LPS) to the peripheral system.

**Figure 1:**
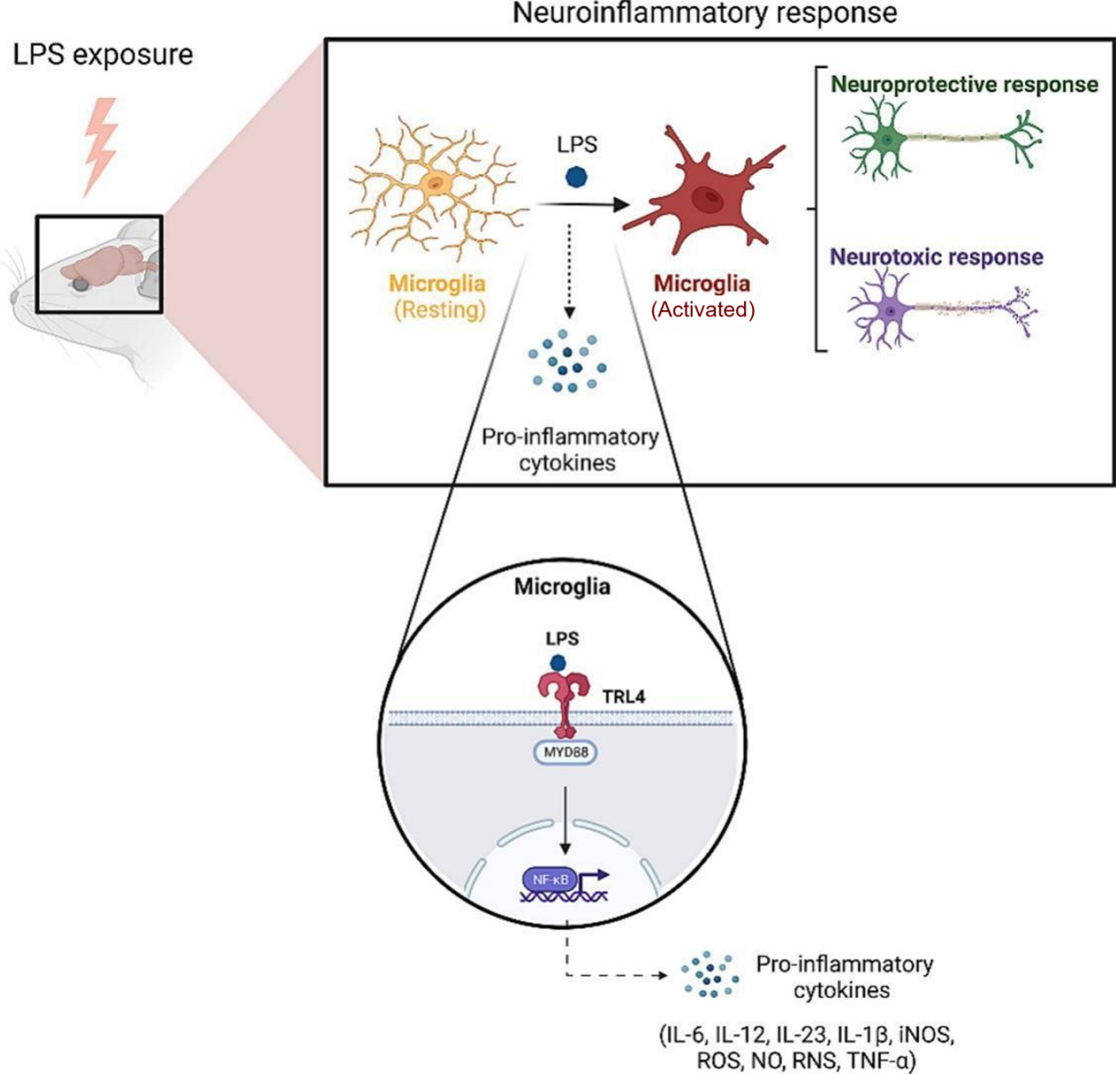
Representation of LPS induced microglial activation. Figure modified from ref (22).

LPS is found in the outer membrane of Gram-negative bacteria and is detected at picomolar levels by toll-like receptor 4 (TLR4). In the periphery, TLR4 is located mostly on immune and nerve cells^13,14^ and with respect to the former, TLR4 are densely expressed on macrophages, monocytes, dendritic cells^15^ and interestingly on Schwann cells: “The neuroglia of the peripheral nervous system”.^16^ LPS has been found to activate NF-κB (an ancient transcription factor that regulates innate immunity)^17^ which induces TNF-α release from Schwann cells.^18^ In macrophages, TLR4 activation triggers biosynthesis of a diverse array of inflammation mediators including TNF-α and IL1-β.^19^ Although LPS does not go through the blood brain barrier (BBB),^20^ TLR4 present in the central nervous system can be reached through the bloodstream due to their localization on microglia (which constitute the BBB) suggesting a vital role for TLR4 in also engaging the innate cerebral immune response.^21^ As part of this response, LPS rapidly activates the microglia,^22^ (Figure 1).

LPS induces rapid sickness behavior in rodents showing that, despite not crossing the BBB, this endotoxin drives behavior^23,24^ While the mechanisms of LPS induced behaviors are not fully established, more details are emerging such as the importance of the vagus nerve in also transducing the LPS induced inflammation signal from the body to the brain.^2^ Interestingly, DA, serotonin, and NE regulate a range of peripheral immune processes, including cytokine secretion, cell adhesion, cytotoxicity, and chemotaxis.^25−29^ Immune system activity can also influence dopaminergic signaling both centrally and peripherally.^30,31^ Serotonin concentration decreases in blood and plasma and accumulates in the liver^32,33^ during LPS-induced inflammation while NE turnover increases in the rat spleen and lungs .^34^

Therefore, it is clear that these monoamines are pivotal in mediating a bidirectional brain/body immune axis. Thus in this perspective, we review the changes in the monoamine concentrations in the extracellular space of the brain in response to LPS and correlate these changes to behavior. It is challenging to measure brain chemistry, and we found studies that included methods such as microdialysis and voltammetry. In the studies, LPS dosage has a big impact on chemical changes. We included only studies that reported measurable impacts on monoamine levels. When accounting for technique artifacts, our interpretation of the literature is that peripheral LPS:

a) decreases DA levels due to increased DA turnover, increased dopamine transporter (DAT) activity and the neurodegenerative effects of LPS on DA neurons.
b) decreases serotonin levels because of increased serotonin turnover, increased serotonin transporter (SERT) activity and increased inhibition of serotonin via H_3_ receptors.
c) increases NE levels because of this messenger’s role in modulating microglial activity.

In sum, having reviewed the effects of peripheral LPS on monoamines, inflammation may serve an important physiological function in altering mood. The sickness/depressed mood may be evolutionarily advantageous to bodily recovery (*i.e*., lethargy forcing rest).^300^

## Dopamine

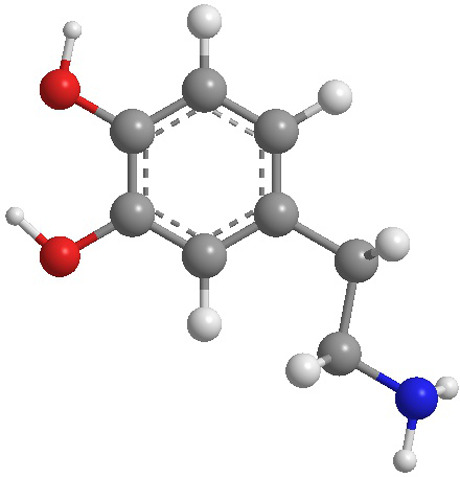



DA is a versatile modulator with various roles in the brain, including cognition and reward. There is much evidence that DA is an envoy of the inflammation message between the brain and body.^35^ For example, hyperdopaminergic systems have been associated with heightened LPS-induced cytokine production in macrophages.^36−38^ In rats, elevating CNS DA levels with L-DOPA affected peripheral T-cells.^39^ DA was found to suppress pro-inflammatory and promote anti-inflammatory cytokines in mouse splenocytes,^40^ and direct activation of dopaminergic neurons in the ventral tegmental area (VTA) of mice, using designer receptors exclusively activated by designer drugs (DREADDs), enhanced the phagocytic activity of splenic dendritic cells and macrophages.^41^ In support of this, Sato et al. showed that repeated stimulation of neurons expressing D1 receptors in the mouse brain nucleus accumbens decreased tumor size in the periphery.^42^

DA is also an important neuromodulator, that has long been implicated in depression. Patients with depression often display motivational and motor impairments and fatigue. These deficits are associated with DA dysfunction in the brain. Several human studies have linked inflammation to reduced motivation, increased fatigue, and motor slowing.^43−48^ One notable study in human volunteers found that after endotoxin injection, there was an increase in self-reported and observer rated depressed mood. These mood states were correlated with altered responsivity in basal ganglia function as measured with fMRI.^49^ Another study showed that treatment with the pro-inflammatory cytokine, IFN-alpha, led to an increase in fatigue and increased glucose metabolism in the basal ganglia (measured with PET), suggesting altered dopaminergic activity.^44^ A final study of note found that humans treated with the typhoid vaccine had increased rates of fatigue that correlated with the proinflammatory cytokine, IL-6 (measured with ELISA). These studies found that humans with higher levels of IL-6 had slower reaction times (motor deficits).^43^ From these studies, it should follow that DA release would be decreased during inflammation and rapidly after LPS. Few people have measured DA chemically after LPS and predominantly with microdialysis, and there are disparate findings with respect to the effect of LPS on DA levels.

Studies tend to agree that the long-term or chronic effect of LPS is a reduction in DA levels, due to LPS’s neurodegenerative effects on DA neurons, increased DA turnover and reduction in DAT activity.^50−54^ However, several studies found that DA levels increased transiently after acute LPS,^53,54^ which is at significant odds with the human behavioral and imaging data and the findings of increased DA turnover.

Some notable studies have shed light on this contradiction. For example, LPS has been shown to induce DAT internalization, which acutely increases DA levels but subsequently reduces DA recycling. This reduction in recycling leads to DA depletion, and when combined with increased DA turnover, ultimately results in decreased DA levels over time.^55,56^ Another study showed that LPS-induced increase in DA was mitigated by indomethacin, a nonsteroidal anti-inflammatory agent.^57^ It is possible that this acute increase in DA arises from local microglia-induced dopaminergic cell death.^58,59^ DA neurons are fragile and do not easily regenerate, in part due to their large energy demand as well as their long, extensive arbors.^60^ It is well documented that microdialysis probe implantation induces immediate activation of microglia.^61,62^ This effect, combined with the fact that LPS also rapidly activates microglia,^63^ is likely to cause local neuronal death around the microdialysis probe. The acute increase in DA may therefore be a consequence of measuring the contents of these dead neurons. Indeed, direct administration of LPS *in vivo* causes microglia-induced loss of dopaminergic neurons.^64,65^

**Figure 2:**
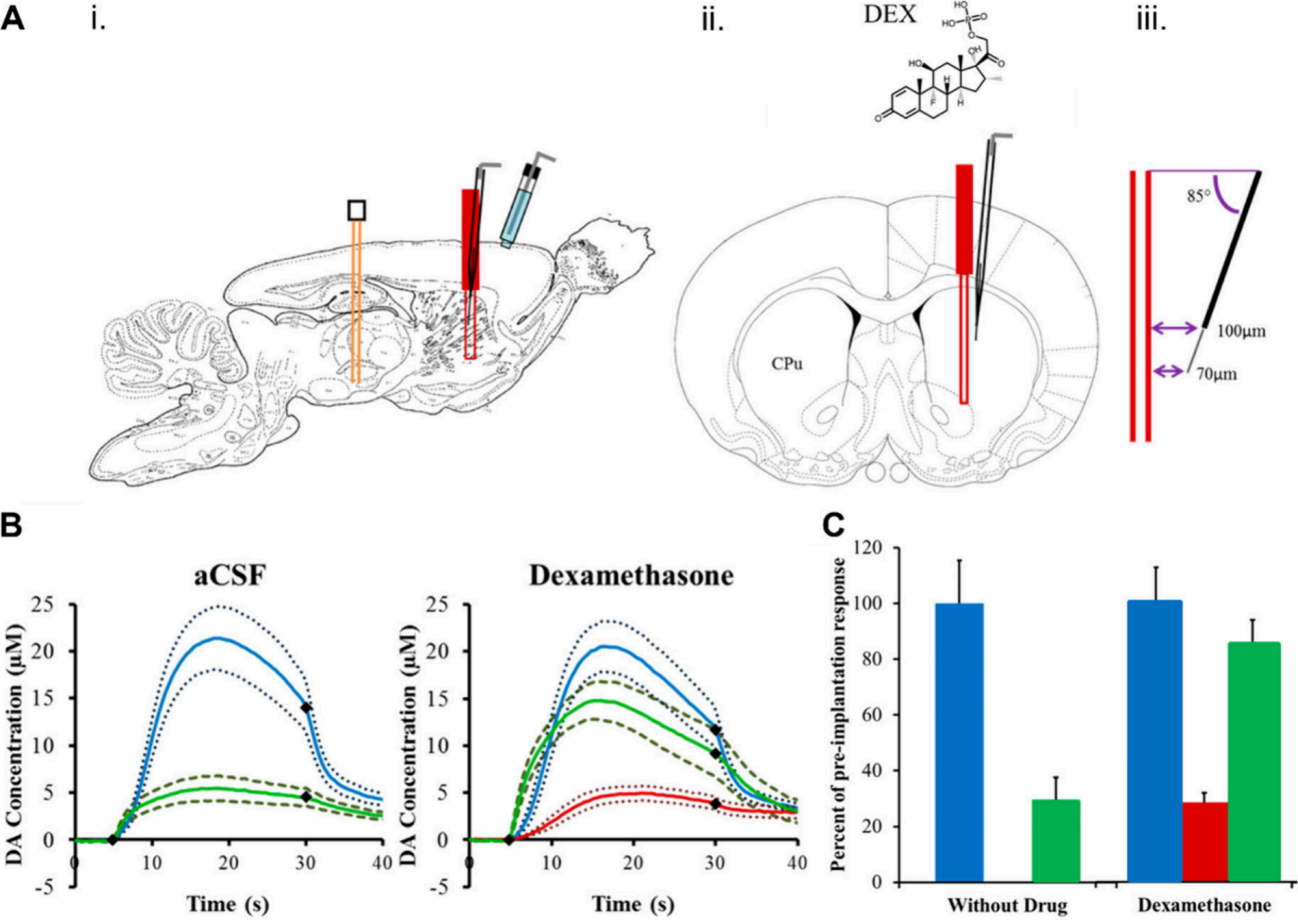
Figure showing (A) experimental setup to test effect of microdialysis probe on DA neurotransmission. (B and C) results showing that the disruption in DA transmission caused by microdialysis probe is rescued with dexamethasone.^62^

In accord with this notion, Nesbitt *et. al* showed, using voltammetry, that the microdialysis probe significantly disrupts dopaminergic transmission, an effect that could be rescued with dexamethasone (glucocorticoid steroid). This rescue effect was hypothesized by the authors to arise from curtailing glial activation with dexamethasone as seen in Figure 2.^62^

In sum, the literature supports different mechanisms leading to reduced DA transmission in response to LPS, that follows the behavioral roles of dopaminergic dysfunction in depression.

## Norepinephrine

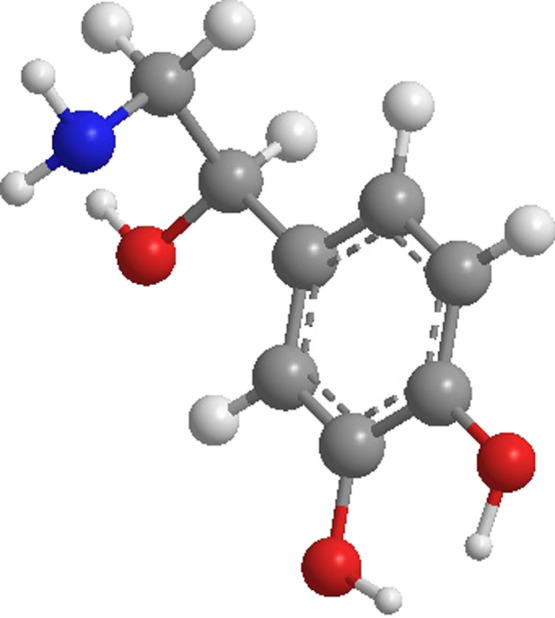



NE, also known as noradrenaline, is thought to be involved in the regulation of mood, attention, and the stress response. NE in the body is produced by the adrenal glands, liver and spleen^66−69^ and plays a strong and well-established role in peripheral inflammation. During inflammation, NE is released from the nerve terminals of lymphoid organs, acting on adrenoreceptors on immune cells.^70^ In this respect, NE has been shown to blunt pro-inflammatory and encourage anti-inflammatory cascades. A subset of intestinal macrophages express β-adrenergic receptors that promote a tissue-protective phenotype,^71^ while adipose macrophages adjacent to sympathetic terminals express functional norepinephrine transporters (NET), which modulate proinflammatory states and thermogenesis.^72^ Stolk et al. showed that in humans under LPS challenge, NE infusion reduced pro-inflammatory cytokines and promoted a better anti-inflammatory cytokine balance.^73^ Furthermore, the group showed that NE inhibited the production of radical oxygen species. This idea was supported in a later review by Thoppil et al., who summarized the literature on how NE regulates oxidative metabolism in immune cells.^74^

**Figure 3:**
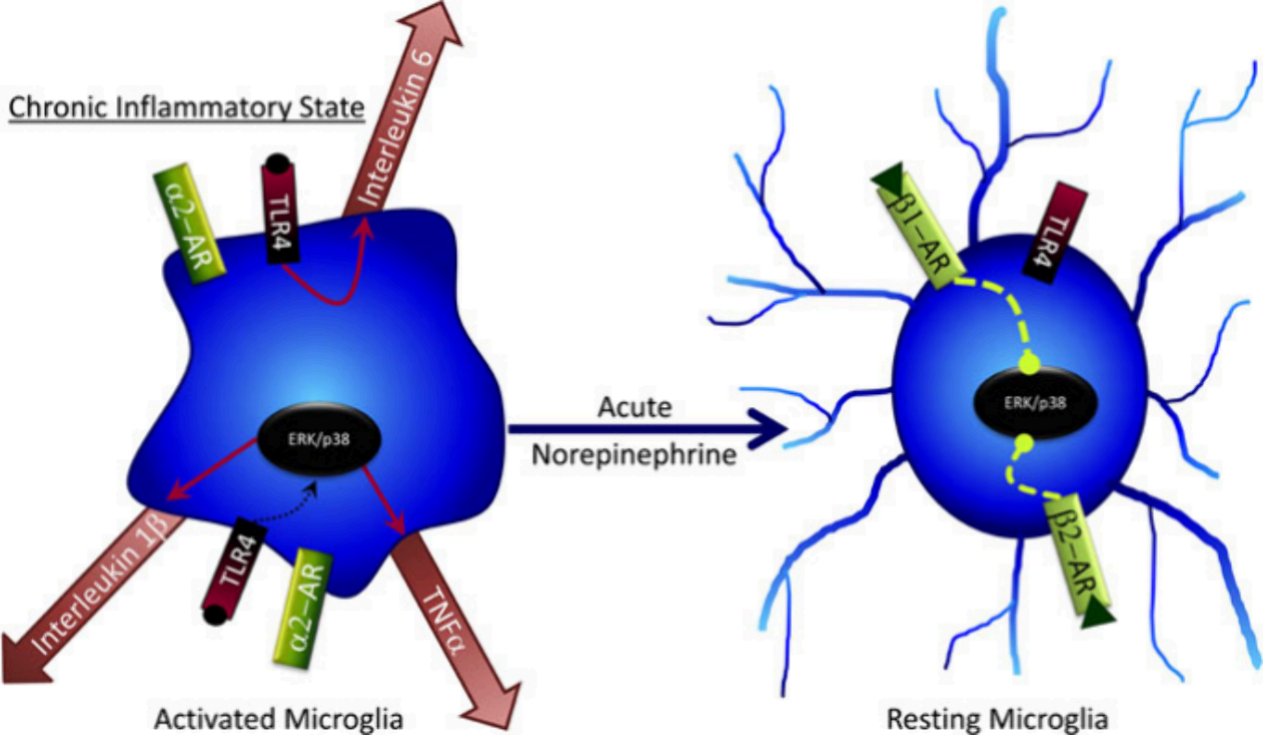
Acute NE exposure can decrease release of cytokines by microglia.^76^

Specifically in the brain during depression, extracellular NE levels are thought to be reduced. This notion has spurred the development of selective NE reuptake inhibitors (NRIs), which are thought to enhance NE availability in the brain by inhibiting this modulator’s reuptake into neurons. However, the premise that depression is associated with decreased NE is not supported by the literature on inflammation and this modulator, indicating a more nuanced relationship. Microdialysis studies above showed that DA increased acutely after LPS, and we attributed this to microglial induced dopaminergic cell death. This sort of cell death may not extend to NE neurons. NE is widely accepted to regulate microglia through β_2_-adrenergic receptor activation.^75^ This receptor activation decreases microglia proliferation and cytokine release, while increasing soma migration and phagocytosis of harmful debris (Figure 3).^76^

Therefore, we propose that in themselves, NE neurons create a chemically privileged microenvironment that makes them less susceptible to microglial induced cell death. In accord with this, *in vitro* tissue slice preparations produce more NE when exposed to LPS and stay viable throughout the experiment.^77^ Indeed, several *in vivo* studies have found NE to increase in several brain structures after LPS.^78,79^

In these studies, the increase in NE is sustained, suggesting a key role for NE during inflammation. Several studies have suggested this role to be neuroprotection of neurons. For example, when rat mesencephalic neuronal cultures were exposed to low doses of NE, the function and survival of the cortical neurons was increased.^80^ Other studies supported this finding by showing that depletion of NE accelerates LPS induced cell death *in vivo*.^81−83^

In sum, it would appear that inflammation rapidly induces a sustained NE response that serves to modulate microglia and protect DA neurons from cell death. We now refine the original clinical notion that NE is reduced in depression since the literature overwhelmingly supports higher NE levels during inflammation. We hypothesize that NRIs do not compensate for a lack of NE but increase NE above baseline.^84^ This increased NE has an anti-inflammatory effect with downstream benefits for depression.

## Serotonin

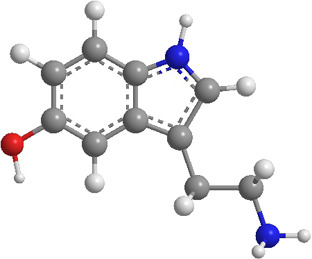



Serotonin is a modulator that is found in high levels in the periphery where it controls digestion, smooth muscle contraction and thermoregulation.^85^ In the body, serotonin is predominantly synthesized by enterochromaffin cells in the gut. It is well-known that serotonin is a potent mediator of peripheral inflammation. With respect to the gut, in particular, there are well-known pathophysiological connections between serotonin and inflammation that cause disorders such as inflammatory bowel disease (IBS).^86−89^ Serotonin receptors and SERTs are expressed on innate immune cells such as monocytes, macrophages, dendritic cells (DCs) and mast cells.^90,91^ During acute inflammation, serotonin stimulates recruitment of some of these immune cells,^92−95^ modifies DC cytokine production, and promotes production of pro-inflammatory cytokines from macrophages.^96−98^ Serotonin also plays an important role in circulatory immunity, where platelets have high concentrations of SERTs.^99−101^ These SERTs take up serotonin into the platelets with high affinity^102^ and release their contents upon immune activation.^103,104^

It is known that peripheral and brain serotonin are connected via the bidirectional vagus immune axis.^105^ Despite only a small fraction of serotonin being present in the brain, this modulator has long been thought to be involved in depression because of the monoamine hypothesis.^6^ This theory states that low extracellular serotonin levels drive the pathology of depression. Selective serotonin reuptake inhibitors (SSRIs) are designed to block the reuptake of serotonin ack into cells, thereby increasing extracellular serotonin levels and alleviating depression symptoms.^106^ SSRIs have variable efficacy; thus, the serotonergic theory of depression has moved in and out of favor for decades, recently enjoying a resurgence because of clinical trials targeting depression with psychedelics (with high affinity for serotonin receptors).^107^ An important recent study found, using positron emission tomography, that serotonin release capacity was reduced in depressed human patients compared with healthy controls.^108^

**Figure 4:**
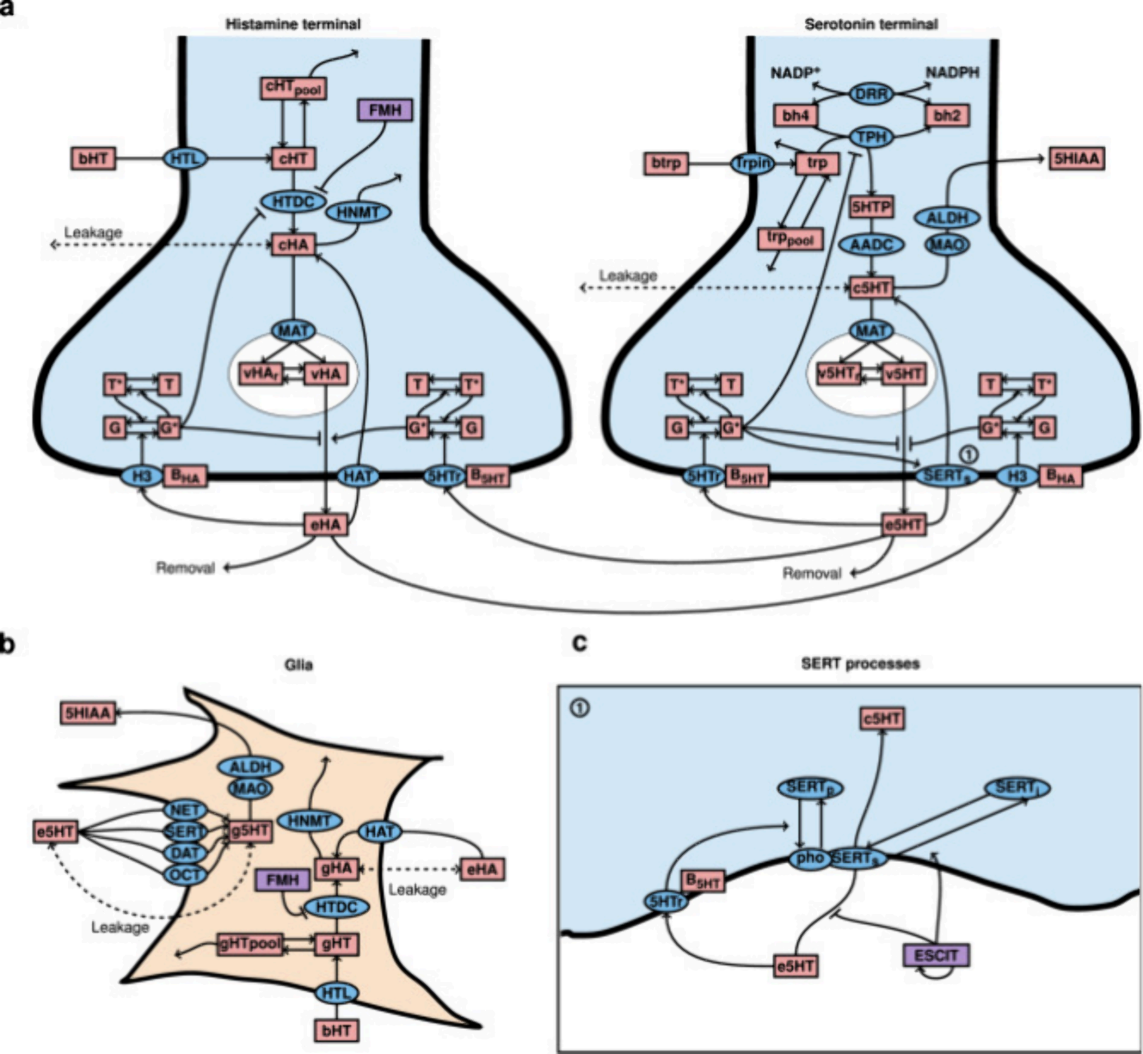
A detailed representation of the comodulation of serotonin, histamine, and glia.^111^

However, contrary to these studies, microdialysis measurements have found serotonin to transiently increase after LPS (as with DA and NE).^53,54,78,79^ A theme in these works is increased turnover or metabolism of serotonin^57^ which theoretically should decrease serotonin levels. Indeed, in our own work with voltammetry, we have shown a rapid decrease in basal hippocampal serotonin after LPS,^109^ which we attributed to activation of inhibitory H_3_ receptors by inflammation induced histamine (Figure 4 shows the biochemical model of histaminergic inhibition of serotonin). This reduction is supported by the theory of increased serotonin turnover and by increased SERT function. In an elegant study by Zhu *et*. *al*, the function of SERTs was shown to rapidly increase after LPS. The authors found that an *i.p*. injection of LPS increased SERT activity through IL-1R and p38 MAPK pathways. Additionally, they showed that systemic LPS induced an increase in immobility during the tail suspension test in mice, indicating despair like behavior, which was not present in SERT knockout mice.^110^

The increase in serotonin observed by microdialysis can be potentially explained via mast cells around the probe. It is well-known that BBB breakage results in rapid mast cell recruitment^112−114^ and mast cell degranulation has been reported at the site of implanted devices.^115^ Mast cells contain serotonin and histamine and release both when activated.^116^ Therefore, we propose that despite LPS induced increased serotonin turnover, increased SERT function and increased inhibition of release via H_3_ receptors, microdialysis observes an increase in serotonin due to serotonin release from mast cells at the microdialysis site.

## Conclusions

Inflammation is synonymous with immune activation and while the effect of inflammation on the body is well researched, there is less known about how peripheral inflammation changes brain chemistry. There is comorbidity between depression and inflammation, thus studying the underlying pathology of inflammation on the brain’s chemicals will aid a better understanding of depression. There is compelling evidence to support the roles of the monoamines DA, serotonin, and NE in both inflammation and depression. In this perspective, we reviewed chemical changes in the brain after LPS, a robust and well-studied way to induce rapid, acute inflammation. From these studies, we interpreted LPS to reduce DA and serotonin and increase NE in the brain. These changes likely serve an important evolutionary purpose (*e.g*., energy conservation for healing).
